# Performance of common primary and chromogenic culture media for MALDI-TOF MS identification of clinically relevant yeasts

**DOI:** 10.1128/spectrum.00974-24

**Published:** 2024-08-20

**Authors:** Simon Lévesque, Nathalie Brown, Philippe J. Dufresne, Catherine Allard

**Affiliations:** 1 Service de microbiologie, CIUSSS de l'Estrie - Centre Hospitalier Universitaire de Sherbrooke, Sherbrooke, Québec, Canada; 2 Département de microbiologie et infectiologie, Faculté de médecine et des sciences de la santé, Université de Sherbrooke, Sherbrooke, Québec, Canada; 3 Laboratoire de santé publique du Québec, Institut national de santé publique du Québec, Sainte-Anne-de-Bellevue, Québec, Canada; University of Chicago, Chicago, Illinois, USA

**Keywords:** MALDI-TOF MS, yeasts, chromogenic media, VITEK-MS, *C. auris*

## Abstract

Timely and accurate identification of yeasts is essential for adequate treatment, considering the increase in antifungal resistance of some species, particularly for *C. auris*. Current matrix-assisted laser desorption/ionization time-of-flight mass spectrometry (MALDI-TOF MS) manufacturer’s protocol for identification of yeasts requires 24- to 72-h cultivation on Sabouraud dextrose media (SAB), but not some of the mainstay primary culture media used in mycology such as inhibitory mold agar (IMA), Mycosel, CHROMagar Candida Plus, and CHROMagar Candida. As culture media can influence MALDI-TOF MS identification results, this study evaluated the accuracy and performance of identification of clinically relevant yeasts on these first-line media using the VITEK-MS MALDI-TOF MS system.

**IMPORTANCE:**

In this study, a panel of 140 strains (21 species) was used to assess the performance of the selected media. Although not in the manufacturer’s list of accepted media, IMA and chromogenic media are suitable for the identification of yeasts on the VITEK-MS systems. CHROMagar Candida Plus allowed the identification of 135/140 isolates tested after 24-h incubation similar to SAB reference media (137/140). Yeast isolates that grew on Mycosel selective media were also reliably identified by matrix-assisted laser desorption/ionization time-of-flight mass spectrometry. VITEK-MS system with IVD database V3.2 correctly identified *C. auris* strains to the species level on CHROMagar Candida Plus alleviating the need for subcultivation and reduced turnaround time (24–72 h) to identification for patient screening.

## INTRODUCTION

Yeast infections and more particularly invasive *Candida* sp. infections have risen significantly in the last two decades (1). Although *C. albicans* is still the main cause of candidiasis, *C. auris* and other *Candida* species are increasingly recovered in patients worldwide. Timely and accurate identification of yeast strains is essential for adequate treatment, especially when one considers the emergence of antifungal resistance of some species and species with intrinsic resistance to some antimycotic agents in use.

Fortunately, the introduction of matrix-assisted laser desorption/ionization time-of-flight mass spectrometry (MALDI-TOF MS) has significantly improved the capacity to correctly identify yeast at species level (2). It has become the mainstay method for the identification of pathogenic microorganisms in clinical laboratories. This system is based on the acquisition of a unique protein signature of each microorganism and its comparison to a well-established and validated MS spectra database. However, growth conditions, particularly the media used, can influence the protein signature and impact the MALDI-TOF MS identification result (3–5). Manufacturers of MALDI-TOF MS systems have a limited set of approved/compatible media for use with their systems, which often does not include some of the common and popular media for isolation of yeasts. The impact on identification performance must be verified for those alternative non-approved media. The majority of studies on MALDI-TOF MS identification of yeasts have been performed on colonies grown on non-selective media and scarce comparative data are available when alternative first-line media, including newly developed chromogenic agars, are used (6–11).

In this study, we evaluated the accuracy and performance of the identification of clinically relevant yeast strains on first-line media using the VITEK-MS MALDI-TOF MS system.

## MATERIALS AND METHODS

A diverse panel of 140 strains (21 species) was used (Table 1; Table S1). All strains had previously been identified using either MALDI-TOF MS using reference media or rRNA sequencing of the ITS and D1D2 regions. The media evaluated in this study were Inhibitory Mold Agar with gentamicin (IMA; Oxoid, Ontario, Canada), BBL Mycosel Agar selective media that contains cycloheximide (MYC; Becton Dickinson, Ontario, Canada), BBL CHROMagar Candida (CC; Becton Dickinson, Ontario, Canada), and CHROMagar Colorex Candida Plus (CCP; Micronostyx, Ontario, Canada). Yeast strains were also grown on Sabouraud Dextrose Agar (SAB; Becton Dickinson, Ontario, Canada; 40 g/L glucose) as comparative reference media for identification of MALDI-TOF MS. The strains were grown at 35°C on each media for 24, 48, and 72 h to obtain sufficient growth. MALDI-TOF MS identification was conducted on the VITEK-MS system (bioMérieux, Marcy-l’Étoile, France) using IVD knowledge database V3.2 with the direct on-slide extraction method with formic acid according to the manufacturer’s instructions. One spot for each strain was deposited on the target slide and only results with an identification score of 99.9% were considered acceptable for a correct identification to the species level, even if the VITEK-MS system considers acceptable an identification score ≥60%.

**TABLE 1:** Correct identification to species obtained with VITEK-MS system (≥99.9% score) for each evaluated media for 140 yeast strains tested

*Candida* species	Number of stains	Sabouraud dextrose (reference)			IMA			Mycosel^*g*^			BBL CHROMagar candida				CHROMagar candida plus		
		24 h	48 h	Total	24 h	48 h	Total	24 h	48 h	Total	24 h	48 h	72 h	Total	24 h	48 h	Total
*Candida albicans*	14	14/14		100%	13/14	1/14	100%	14/14		100%	14/14			100%	14/14		100%
*Candida auris*	19	18/19	1/19	100%	19/19		100%			N/A	14/19	3/19	2/19	100%	18/19	1/19	100%
*Candida dubliniensis*	5	5/5		100%	5/5		100%	4/5	1/5	100%	4/5	0/1		80%	2/5	0/5	40%
*Candida duobushaemuli*	3	3/3		100%	3/3		100%			N/A	2/3	1/3		100%	3/3		100%
*Candida glabrata*	44	42/44	2/44	100%	41/44	3/44	100%	1/1		100%	34/44	10/44		100%	44/44		100%
*Candida guilliermondii^a^*	3	3/3		100%	3/3		100%	1/3	2/3	100%	3/3			100%	3/3		100%
*Candida haemuli*	3	3/3		100%	3/3		100%			N/A		3/3		100%	3/3		100%
*Candida intermedia*	1	1/1		100%	1/1		100%			N/A		1/1		100%	1/1		100%
*Candida kefyr^b^*	1	1/1		100%	1/1		100%	1/1		100%	1/1			100%	1/1		100%
*Candida krusei^c^*	5	5/5		100%	5/5		100%			N/A	5/5			100%	5/5		100%
*Candida lusitaniae^d^*	3	3/3		100%	3/3		100%			N/A	2/3		1/3	100%	2/3	1/3	100%
*Candida parapsilosis*	15	15/15		100%	6/15	9/15	100%			N/A	8/15	5/15	2/15	100%	15/15		100%
*Candida pararugosa^e^*	1	1/1		100%	1/1		100%			N/A		1/1		100%	1/1		100%
*Candida pelliculosa^f^*	1	1/1		100%	1/1		100%			N/A	1/1			100%	1/1		100%
*Candida tropicalis*	5	5/5		100%	4/4	1/1	100%		5/5	100%	4/5	1/5		100%	5/5		100%
*Cryptococcus neoformans*	6	6/6		100%	6/6		100%			N/A	6/6			100%	6/6		100%
*Cryptococcus gattii*	1	1/1		100%	1/1		100%			N/A	1/1			100%	1/1		100%
*Kodamea ohmeri*	1	1/1		100%	1/1		100%			N/A	1/1			100%	1/1		100%
*Rododula mucilaginosa*	3	3/3		100%	3/3		100%			N/A	3/3			100%	3/3		100%
*Saccharomyces cerevisiae*	3	3/3		100%	3/3		100%			N/A	1/3	2/3		100%	3/3		100%
*Trichosporon asahii*	3	3/3		100%	3/3		100%		3/3	100%	3/3			100%	3/3		100%
Total	140	137/140 (98%)	3/140	100%	126/140 (90%)	14/140	100%	21/32 (66%)	11/32	100%	107/140 (76%)	27/140	5/140	99.3%	135/140 (96%)	2/140	97.9%

^*a*^ *Meyerozyma guilliermondii.*

^*b*^ *Kluyveromyces marxianus.*

^*c*^ *Pichia kudriavzevii.*

^*d*^ *Clavispora lusitaniae.*

^*e*^ *Wickerhamiella pararugosa.*

^*f*^ *Wickerhamomyces anomalus.*

^*g*^ The results are limited to the 19 strains which grew out of the 140 strains plated on Mycosel selective media.

## RESULTS

**Fig 1:**
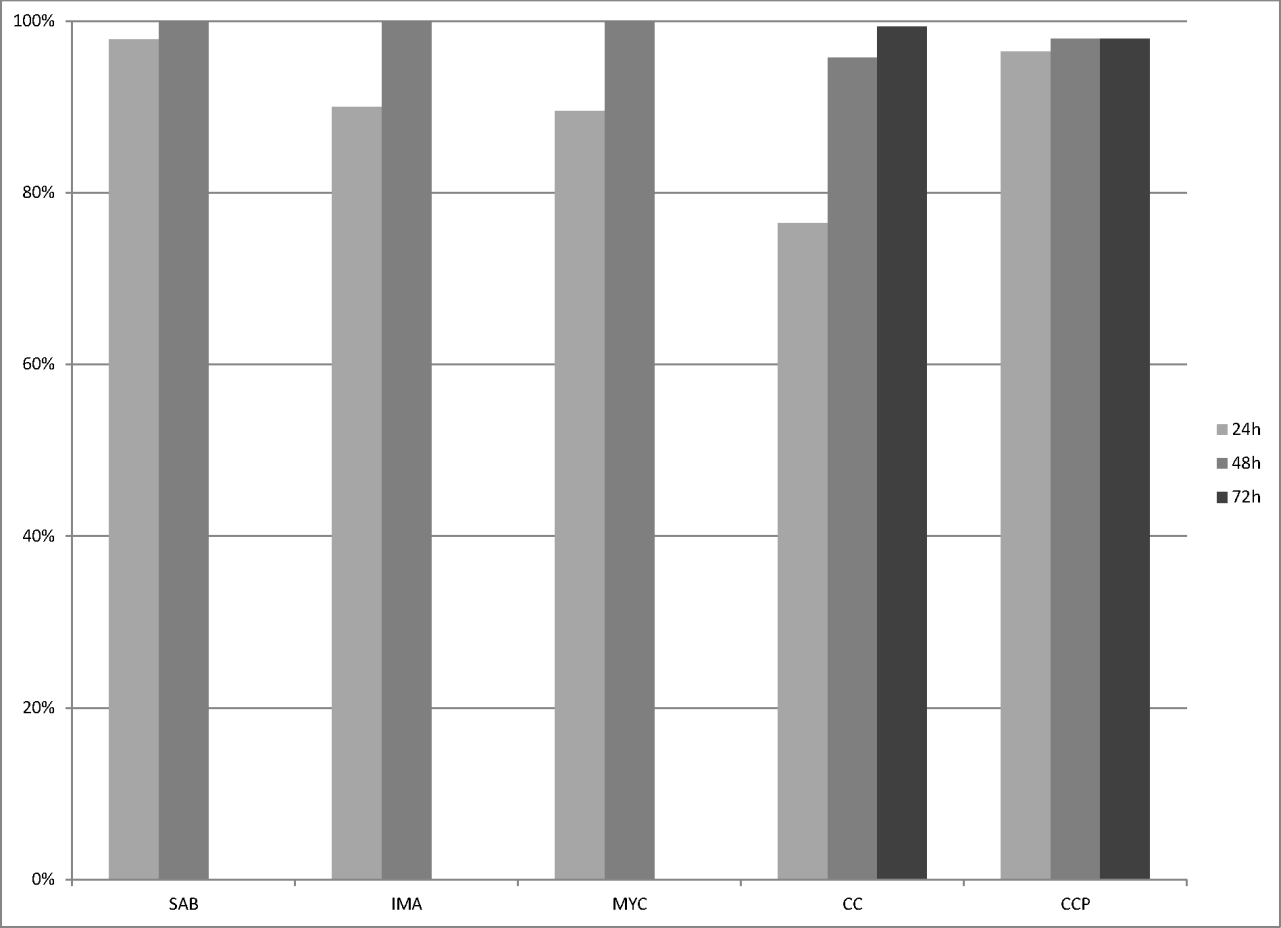
Percentage of correct MALDI-TOF MS identification of *Candida* sp. and other yeast of clinical importance strains (*n* = 140) after 24 h, 48 h, or 72 h of incubation on the evaluated media. Results for MYC media are for the 32 strains that grew on this medium.

Table 1 shows the results obtained for each media analyzed. SAB, IMA, and MYC gave 100% correct species identification, achieved with variable incubation time. CC and CCP gave 99.3% and 97.9% of correct species identification, respectively. One strain of *C. dubliniensis* was misidentified as *C. albicans* on CC and three strains of *C. dubliniensis* were misidentified as *C. albicans* on CCP. The four misidentifications resulted in a score of 99.9% for *C. albicans*. Except for MYC media, all strains were able to grow after only 24 h. However, out of the 140 strains tested, prolonged incubation time was necessary to obtain a correct identification for 3, 14, 27, and 2 strains with SAB, IMA, CC, and CCP media, respectively. For five strains (two *C*. *auris*, two *C*. *parapsilosis*, and one *C*. *lusitaniae*), 72 h of incubation was necessary to obtain an adequate identification on CC (Fig. 1). All the strains with prolonged incubation time (more than 24 h) gave “No identification” results on the first identification attempt. Overall, only 32 strains grew on MYC media, most being species known capable of growth on MYC (*C. albicans, C. dubliniensis*, *C. guilliermondii, C. kefyr, C. tropicalis,* and *T. asahii*) with the exception of 1 *C. glabrata* isolate. Twenty-one (21) out of those 32 isolates (65.6%) could be reliably identified after 24 h.

## DISCUSSION

To our knowledge, the evaluated common mycology culture media tested in our study are not supported by bioMérieux as suitable for VITEK-MS identification (12). As many media are available for the primary inoculation and cultivation of fungi from clinical specimens, it is essential to verify if the selected media are suitable for MALDI-TOF MS identification and give an accurate identification. Performing MALDI-TOF MS identification directly on the primary culture saves time and cost as it avoids the need for subcultivation. Of note, the performance of the recently launched CHROMagar Candida Plus, useful for *C. auris* screening, is excellent and comparable to the SAB media with 96.4% correct identification after 24 h of growth, alleviating the need to recultivate characteristic aqua colonies with blue halo that are presumptive *C. auris*. In this study, we chose to use an identification score of 99.9% as a good identification to the species level. Although the manufacturer accepts ≥60% as a good identification score, local experiments (not only for yeast but also for bacteria) have demonstrated that misidentification can occur below a score of 99.9% (data not shown).

Only two studies previously evaluated CCP performance for MALDI-TOF MS identification but on the Bruker biotyper system (10, 13). Our study shows that CCP also performs well in the VITEK-MS system. Other major clinical *Candida* species such as *C. albicans*, *C. tropicalis*, *C. glabrata,* and *C. krusei* were also identified reliably with CCP agar. Other studies that evaluated time of growth did not observe significant discrepancy in the percentage of correct identification according to time (7, 10, 11); however, they could not reach 100% of correct identification to species with all of the evaluated strains. In our study, only four misidentifications (2.8%) were obtained, all of which resulted from *C. albicans/C. dubliniensis* identification inversions on CP and CCP agar. The clinical impact of those erroneous identifications could likely be controlled by reporting those two closely related species at the species complex when those chromogenic agars were used. In all other cases, prolonged incubation was necessary due to no identification or low score identification results at 24 h of growth.

This study has some limitations. First, we recognize the limited assessment of the MYC media, since only 32 of the 140 strains included in the study were evaluated. Further studies with more strains of those species that grow on MYC are needed to truly assess the impact this agar could have on identification by MALDI-TOF MS. Second, this study was limited to VITEK-MS system and other studies using the Bruker system or the new VITEK-MS PRIME system should be performed to confirm the performance of the evaluated media. Third, all the strains were cultured at 35°C. Yeast cultivation from primary specimens is usually performed at 30°C, but CHROMagar manufacturer recommendation is 35°C. We do not observe a particular growth effect between both incubation temperatures in our laboratory.

In conclusion, we demonstrated in this study that IMA, MYC, CC, and CCP are suitable media for the direct identification of clinically relevant yeast species with the VITEK-MS system. Best turnaround time was obtained with SAB reference media and CCP with 97.8% and 96.4% identification rate after 24 h, while IMA, MYC, and CC can require extended incubation up to 72 h for identification. This highlights the compatibility of the most common culture media for identification by MALDI-TOF MS. In addition, it may be advantageous for laboratories to perform subcultures using approved media if yeast is not identified after 24 h using unapproved media, to ensure that identification is achieved in a timely manner. However, there is a definite need in a clinical diagnostic setting to validate any media that is not endorsed as compatible by MALDI-TOF MS manufacturers to avoid identification delays, failure to identify, and potentially misidentification of microorganisms. The variable growth and various protein signatures generated on different media must be carefully evaluated. Some of the issues reported for species-media combination could likely be alleviated by the creation of custom superspectra (through SARAMIS database available for VITEK-MS system) of those organisms in these culture conditions and ideally added to the manufacturer’s reference spectra and list of accepted cultivation media.

## SUPPLEMENTAL MATERIAL

Table S1 spectrum.00974-24-s0001.xlsx List of strains used in the study (n=140).

ASM does not own the copyrights to Supplemental Material that may be linked to, or accessed through, an article. The authors have granted ASM a non-exclusive, world-wide license to publish the Supplemental Material files. Please contact the corresponding author directly for reuse.
